# Prevalence of vasectomy and tubal ligation: time series analysis Brazil, 2006–2019

**DOI:** 10.1590/S2237-96222026v35e20240474.en

**Published:** 2025-12-19

**Authors:** Patrícia Fernandes Murta, Fernanda Gontijo Araújo, Torcata Amorim, Gisele Nepomuceno de Andrade, Mariana Santos Felisbino-Mendes

**Affiliations:** 1 Universidade Federal de Minas Gerais, Belo Horizonte, MG, Brasil

**Keywords:** Vasectomy, Sterilization, Tubal, Family Development Planning, Health Status Disparities, Health Surveys, Vasectomía, Esterilización Tubaria, Planificación Familiar, Disparidades en el Estado de Salud, Encuestas Epidemiológicas.

## Abstract

**Objective:**

To analyze the temporal trends in the prevalence of vasectomy and tubal ligation in 2006, 2013, and 2019 according to sociodemographic characteristics of the Brazilian population of reproductive age.

**Methods:**

A time series analysis was conducted using secondary data from three comparable national surveys that investigated the reproductive planning of Brazilian women. The prevalence of partner-reported vasectomy and tubal ligation was estimated with 95% confidence intervals.

**Results:**

An increase in the prevalence of partners’ vasectomy was observed, from 4.2% to 5.6% among all women, and from 5.2% to 7.8% among those living with a partner, between 2013 and 2019. A decrease in tubal ligation prevalence was observed, from 32.2% to 17.3% among all women, and from 36.3% to 21.8% among those living with a partner, between 2006 and 2019. Black and Brown (Brazilian mixed race) women (2006 – 37.1%; 2013 – 27.2%; 2019 – 20.6%), those who had attended elementary school (2006 – 43.4%; 2013 – 36.8%; 2019 – 30.8%), living in rural areas (2006 – 40.0%; 2013 – 33.0%; 2019 – 26.8%), and who had three births (2006 – 64.2%; 2013 – 54.0%; 2019 – 44.1%) showed higher tubal ligation prevalence throughout the analyzed period. Women with private health insurance (2006 – 9.0%; 2013 – 6.2%; 2019 – 8.6%) reported higher vasectomy prevalence among their partners.

**Conclusion:**

Despite the progress observed through the increased prevalence of vasectomy and the reduction of tubal ligation, gender and social inequalities regarding the use of these methods persist, highlighting the need to expand equitable access to long-acting contraceptive methods in the country.

## Resumen

**Objetivo:**

Analizar la evolución temporal de la prevalencia de vasectomía y ligadura de trompas en 2006, 2013 y 2019 según las características sociodemográficas de la población brasileña en edad reproductiva.

**Métodos:**

Análisis de serie temporal que utilizó datos secundarios de tres encuestas nacionales comparables que investigaron la planificación reproductiva de las mujeres brasileñas. Se estimó la prevalencia de vasectomía de los compañeros referida por las mujeres y de ligadura de trompas, con intervalos de confianza del 95%.

**Resultados:**

Se observó un aumento en la prevalencia de vasectomía de los compañeros, del 4,2% al 5,6% entre todas las mujeres y del 5,2% al 7,8% entre las que vivían con su pareja entre 2013 y 2019. La prevalencia de ligadura de trompas disminuyó del 32,2% al 17,3% entre todas las mujeres y del 36,3% al 21,8% entre las que vivían con su pareja entre 2006 y 2019. Las mujeres negras y pardas (personas de ascendencia mixta) (2006 – 37,1%; 2013 – 27,2%; 2019 – 20,6%), con educación primaria (2006 – 43,4%; 2013 – 36,8%; 2019 – 30,8%), residentes en áreas rurales (2006 – 40,0%; 2013 – 33,0%; 2019 – 26,8%) y con tres partos (2006 – 64,2%; 2013 – 54,0%; 2019 – 44,1%) presentaron mayores prevalencias de ligadura de trompas en todo el período analizado. Las mujeres con seguro de salud (2006 – 9,0%; 2013 – 6,2%; 2019 – 8,6%) reportaron mayores prevalencias de vasectomía en sus parejas.

**Conclusión:**

A pesar de los avances observados con el aumento de la prevalencia de vasectomía y la reducción de la ligadura de trompas, persisten desigualdades de género y sociales en el uso de estos métodos, lo que señala la necesidad de ampliar el acceso equitativo a los métodos anticonceptivos de larga duración en el país.

**Ethical aspects**

This research used publicly available and anonymized databases.

## Introduction 

Vasectomy and tubal ligation, permanent contraceptive methods, are invasive surgical procedures used by individuals and couples who do not wish to have children or have already reached their desired number of children (1). While tubal ligation is one of the most widely used methods worldwide, with approximately 19% of users, vasectomy is used by less than 3% of men (1).

In Brazil, during the 1980s, tubal ligation became widespread, being performed mainly among low-income women and in poorer regions (2). In 1983, the Program for Integral Assistance to Women’s Health (*Programa de Assistência Integral à Saúde da Mulher*, PAISM) proposed actions to promote and prevent women’s health and to support reproductive planning (3). However, these actions emphasized tubal ligation as a highly effective contraceptive method, often performed during cesarean section (4) to cover its costs, as it was not eligible for reimbursement by the public health system (5). 

This contraceptive method became the main contraceptive choice among couples (6), increasing the proportion of sterilized women who were married or living with a partner, from 27% in 1986 to 40% in 1996 (7,8). In 1986, 75% of sterilizations performed in the country took place during cesarean sections (9). Tubal ligation was also provided free of charge as a political favor, mainly in the Northeast region (5). 

In 1992, a Parliamentary Inquiry Commission (*Comissão Parlamentar de Inquérito*, CPI) investigated this reality in the country and published a report highlighting the growing demand for contraception and the limited availability of contraceptive methods, particularly among low-income populations (6). As a result, in 1996, the Family Planning Act (*Lei do Planejamento Familiar*) was enacted. This public policy aimed to regulate surgical sterilization and expand access to other contraceptive methods within the framework of the Unified Health System (*Sistema Único de Saúde*, SUS) (10). Only in 2022 was this act revised, allowing men and women aged over 21 years to undergo surgical sterilization, including during birth (11).

Permanent contraception is important for women, especially in a context where access to other methods with similar effectiveness, such as the intrauterine device, is still limited and not widely available. In Brazil, long-acting reversible contraceptives are more frequently used by women with private health insurance, higher income, and higher education level, whereas permanent methods are more commonly used by women with lower education levels (12). This situation reveals inequities in the use of contraceptive methods in Brazil (12,13). 

Most studies address sterilization as a whole or focus exclusively on tubal ligation (5,6,9,12), which prevents the monitoring of trends for each type of sterilization in the country. They also limit the assessment of these methods to women living with a partner (5,6,9), highlighting the need to evaluate separately the temporal trends in the use of these contraceptive methods in Brazil. 

This study aimed to analyze the temporal trends in the prevalence of vasectomy and tubal ligation in Brazil in 2006, 2013, and 2019, according to sociodemographic characteristics of the population of reproductive age. 

## Methods 

### Study design and setting

This is an epidemiological time series study using secondary data from Brazilian women of reproductive age who answered questions on reproductive planning in national health surveys.

### Study population

For this analysis, Brazilian women of reproductive age who were users of contraceptive methods and were not pregnant were considered. This population was subdivided into two groups: all women and those living with a partner. This procedure was applied in all three surveys.

### Data sources and study size

Three population surveys were used: the Brazilian National Demographic and Health Survey of Children and Women (*Pesquisa Nacional de Demografia e Saúde da Criança e da Mulher*, PNDS) 2006, the National Health Survey (*Pesquisa Nacional de Saúde*, PNS) 2013, and the PNS 2019.

The 2006 PNDS was a national household survey conducted using a complex, two-stage probabilistic sampling design. The first stage comprised the primary sampling units, represented by census tracts. The second stage consisted of the secondary sampling units, represented by households. The sample included private households and communities, selected across 10 strata for the five macroregions of the country (North, Northeast, South, Southeast, and Central-West), covering both urban and rural areas. A total of 14,617 households were selected, and 15,575 women aged 15 to 49 years were interviewed (14,15). 

The PNS, a national household survey, was conducted in 2013 and 2019, with samples corresponding to a subsample of the Master Sample of the Integrated System of Household Surveys (*Sistema Integrado de Pesquisas Domiciliares*, SIPD) of the Brazilian Institute of Geography and Statistics (*Instituto Brasileiro de Geografia e Estatística*, IBGE). The Master Sample consisted of a set of primary sampling units from selected areas, designed to support surveys within the Integrated System of Household Surveys (SIPD). These primary sampling units were used in the PNS sampling design. 

The 2013 PNS sample was designed in three stages: selection of a subsample of primary sampling units in each stratum of the Master Sample with probability proportional to size; simple random sampling of households within each selected primary sampling unit; and simple random selection of one individual aged 18 years or older among all adult residents of the household (16). A total of 63,348 households were selected, and 22,621 women aged 18 to 49 years were interviewed in 2013 (13,17). 

The 2019 PNS sample followed the same methodology; however, interviewees were required to be aged 15 years or older (18). A total of 94,114 households were selected, and 27,249 women of reproductive age were interviewed (12,19). 

### Variables 

The outcome was the use of permanent contraception as reported by women of reproductive age, which included vasectomy by the partner and tubal ligation by the woman. 

The explanatory variables were the sociodemographic characteristics of the women. Age group was categorized as 15–19 years for the 2006 PNDS and the 2019 PNS, and 18–19 years for the 2013 PNS. The other age categories were the same across the three surveys: 20–24, 25–29, 30–34, 40–44, and 45–49 years. The remaining variables were categorized as follows: race/skin color (White, Black/Brown [Brazilian mixed race]); education level (elementary school, high school, higher education); region of residence (Central-West, North, Northeast, Southeast, South); place of residence (urban or rural area); private health insurance (no, yes); and number of childbirths (none, one, two, three, four or more). The Asian and Indigenous categories were excluded due to the very small sample size, which did not ensure representativeness of these population groups. 

### Bias control

Partner-reported vasectomy could result in lower observed prevalence of this method, since women—particularly those who are single—might not know whether their partners had undergone the procedure. Differences in age groups could also hinder comparability, particularly because the 2013 PNS did not include women aged 15–17 years in its sampling design.

### Statistical methods

The prevalence of partner-reported vasectomy and tubal ligation was estimated, considering in the numerator the users of these methods and in the denominator all women using any contraceptive method. Next, the prevalence of each permanent contraceptive method was estimated according to sociodemographic characteristics. All estimates were calculated with 95% confidence intervals (95%CI) to analyze differences between groups. 

Data were analyzed using the Stata statistical software, survey module, to obtain population estimates, considering stratum, cluster, and individual weights. The weights of the 2013 PNS dataset were adjusted to enable comparison with the 2019 survey (20).

## Results 

**Figure 1:**
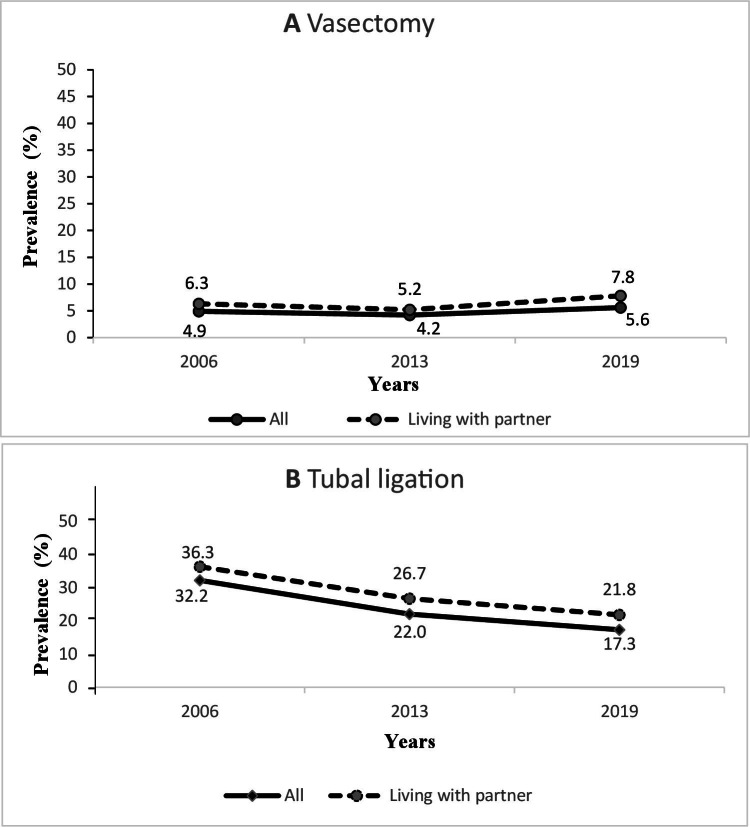
Temporal trend in prevalence: (A) partner-reported vasectomy; (B) tubal ligation. Brazil, 2006-2019

An increase in the prevalence of partner-reported vasectomy among Brazilian women of reproductive age was observed between 2013 and 2019, from 4.2% to 5.6% (Table 1)—an increase of 33.3%, which was even higher among women living with a partner, from 5.2% to 7.8% (Table 2; Figure 1A). The prevalence of tubal ligation was 32.2% in 2006 and 17.3% in 2019, representing a 46.3% decrease. For women living with a partner, this reduction was 39.9%, ranging from 36.3% in 2006 to 21.8% in 2019 (Figure 1B).

**Table 2:** Prevalence (%) and 95% confidence interval (95%CI) of partner vasectomy reported by Brazilian women of reproductive age living with a partner, according to sociodemographic characteristics. Brazil, 2006, 2013, and 2019

Sociodemographic characteristics	2006^a^	2013^b^	2019^c^
	%^d^ (95%CI)	%^d^ (95%CI)	%^d^ (95%CI)
Total	6.3 (5.4; 7.4)	5.2 (4.5; 6.1)	7.8 (7.0; 8.8)
**Age group** (years)			
15–19^e^	0.9 (0.2; 3.3)	-	0.3 (0.0; 2.2)
18–19^f^	-	0.0 (0.0; 0.7)	-
20–24	0.5 (0.2; 1.4)	0.4 (0.1; 1.1)	0.4 (0.2; 1.1)
25–29	4.2 (2.4; 7.3)	2.9 (1.7; 4.8)	2.6 (1.6; 4.2)
30–34	8.8 (6.3; 12.1)	5.4 (4.0; 7.3)	4.7 (3.4; 6.6)
35–39	7.5 (5.4; 10.4)	5.7 (4.3; 7.6)	11.4 (9.4; 13.7)
40–44	10.0 (7.4; 13.5)	10.0 (7.7; 12.9)	11.6 (9.3; 14.4)
45–49	6.8 (4.7; 9.8)	6.4 (4.3; 9.4)	13.6 (10.6; 17.3)
**Race/skin color**			
White	8.4 (6.8; 10.2)	6.6 (5.4; 8.1)	9.0 (7.7; 10.6)
Black/Brown	4.8 (3.7; 6.2)	4.0 (3.2; 5.1)	7.0 (6.0; 8.2)
**Education Level**			
Elementary School	4.9 (3.8; 6.3)	3.8 (2.8; 5.0)	6.5 (5.0; 8.4)
High School	6.5 (5.0; 8.4)	6.0 (4.8; 7.4)	7.7 (6.5; 9.2)
Higher Education	12.8 (9.5; 17.1)	6.7 (5.1; 8.7)	9.6 (8.0; 11.5)
Region			
Central-West	4.7 (3.4; 6.2)	5.8 (4.3; 7.8)	7.7 (5.8; 10.1)
North	0.9 (0.5; 1.8)	1.4 (0.7; 2.5)	1.8 (1.3; 2.7)
Northeast	1.8 (1.1; 2.9)	2.5 (1.6; 3.8)	2.6 (2.0; 3.5)
Southeast	10.3 (8.3; 12.7)	7.8 (6.2; 9.6)	12.1 (10.3; 14.2)
South	6.6 (4.9; 8.7)	5.4 (4.0; 7.2)	8.7 (6.9; 10.8)
**Place of residence**			
Urban Area	7.2 (6.1; 8.4)	5.9 (5.0; 6.8)	8.7 (7.7; 9.7)
Rural Area	2.5 (1.1; 5.9)	1.8 (1.1; 3.0)	3.2 (2.2; 4.5)
**Health Insurance**			
No	4.2 (3.3; 5.2)	4.0 (3.2; 4.8)	6.1 (5.2; 7.2)
Yes	12.1 (9.7; 15.0)	8.2 (6.7; 10.1)	12.0 (10.2; 14.1)
Parity			
None	6.0 (1.1; 26.4)	0.4 (0.0; 3.0)	5.9 (1.5; 19.8)
1	2.7 (1.8; 4.1)	2.2 (1.4; 3.4)	4.0 (3.0; 5.4)
2	9.9 (8.1; 12.0)	8.0 (6.5; 9.7)	12.4 (10.7; 14.3)
3	7.2 (4.9; 10.5)	6.7 (4.8; 9.2)	9.6 (7.3; 12.5)
4+	4.0 (2.4; 6.5)	4.1 (2.9; 6.0)	8.1 (5.1; 12.5)

^a^Results based on data from the 2006 Brazilian National Demographic and Health Survey of Children and Women (PNDS) (n=8,023); ^b^Results based on data from the 2013 National Health Survey (PNS) (n=9,994); ^c^Results based on data from the 2019 PNS (n=10,744); ^d^Population estimates; ^e^Age group used for analyses with data from the 2006 PNDS and 2019 PNS; ^f^Age group used with data from the 2013 PNS.

**Table 1:** Prevalence (%) and 95% confidence interval (95%CI) of partner-reported vasectomy among all Brazilian women of reproductive age, according to sociodemographic characteristics. Brazil, 2006, 2013, and 2019

Sociodemographic characteristics	2006^a^	2013^b^	2019^c^
	%^d^ (95%CI)	%^d^ (95%CI)	%^d^ (95%CI)
Total	4.9 (4.1; 5.7)	4.2 (3.6; 4.8)	5.6 (5.0; 6.2)
**Age group** (years)			
15–19^e^	0.4 (0.0; 1.5)	-	0.4 (0.1; 1.1)
18–19^f^	-	0.0 (0.0; 1.0)	-
20–24	0.4 (0.2; 0.9)	0.3 (0.1; 0.7)	0.2 (0.1; 0.5)
25–29	3.2 (1.9; 5.5)	2.3 (1.4; 3.7)	2.0 (1.3; 3.1)
30–34	7.3 (5.3; 10.1)	4.4 (3.3; 5.9)	3.8 (2.7; 5.2)
35–39	7.0 (5.2; 9.5)	5.1 (3.8; 6.8)	9.1 (7.6; 10.9)
40–44	8.5 (6.2; 11.4)	9.0 (7.0; 11.5)	9.5 (7.7; 11.7)
45–49	5.4 (3.7; 7.8)	6.4 (4.5; 9.1)	11.8 (9.3; 14.8)
**Race/skin color**			
White	6.4 (5.3; 7.8)	5.2 (4.3; 6.3)	6.3 (5.4; 7.4)
Black/Brown	3.7 (2.9; 4.9)	3.2 (2.6; 4.0)	5.1 (4.4; 5.9)
**Education Level**			
Elementary School	4.1 (3.2; 5.3)	3.5 (2.7; 4.5)	5.3 (4.1; 6.7)
High School	4.6 (5.6; 5.9)	4.5 (3.7; 5.6)	5.3 (4.5; 6.3)
Higher Education	8.0 (6.0; 10.7)	4.6 (3.5; 6.0)	6.2 (5.2; 7.4)
Region			
Central-West	3.9 (3.0; 5.3)	4.6 (3.5; 6.1)	5.8 (4.5; 7.5)
North	0.8 (0.4; 1.6)	1.2 (0.7; 2.0)	1.8 (1.2; 2.5)
Northeast	1.5 (0.9; 2.4)	2.2 (1.4; 3.5)	2.1 (1.6; 2.6)
Southeast	7.5 (6.1; 9.2)	5.7 (4.7; 7.0)	8.1 (6.9; 9.5)
South	5.1 (3.8; 6.8)	4.5 (0.3; 6.0)	6.4 (5.2; 8.0)
**Place of residence**			
Urban Area	5.3 (4.6; 6.3)	4.5 (3.9; 5.2)	5.9 (5.3; 6.7)
Rural Area	2.2 (0.9; 5.0)	1.8 (1.1; 2.8)	2.9 (2.1; 4.0)
**Health Insurance**			
No	3.2 (2.6; 4.0)	3.2 (2.7; 3.9)	4.4 (3.8; 5.1)
Yes	9.0 (7.2; 11.2)	6.2 (5.1; 7.6)	8.6 (7.3; 10.1)
Parity			
None	4.9 (1.2; 18.5)	0.5 (0.1; 1.8)	3.4 (1.2; 9.6)
1	2.4 (1.6; 3.5)	1.9 (1.3; 2.9)	3.3 (2.5; 4.3)
2	9.0 (7.4; 10.9)	7.0 (5.7; 8.6)	10.5 (9.1; 12.1)
3	6.2 (4.2; 9.1)	6.3 (4.6; 8.6)	8.4 (6.5; 10.7)
4+	2.1 (1.2; 3.5)	2.8 (2.0; 3.8)	6.6 (4.3; 10.1)

^a^Results based on data from the 2006 Brazilian National Demographic and Health Survey of Children and Women (PNDS) (n=11,324); ^b^Results based on data from the 2013 National Health Survey (PNS) (n=14,394); ^c^Results based on data from the 2019 PNS (n=16,640); ^d^Population estimates; ^e^Age group used for analyses with data from the 2006 PNDS and 2019 PNS; ^f^Age group used with data from the 2013 PNS.

An increase in partner-reported vasectomy was observed among all women aged 45 years and older, from 5.4% in 2006 to 11.8% in 2019 (Table 1). This was also observed among women living with a partner (6.8% to 13.6%) (Table 2). An increase in partner-reported vasectomy was noted for all women who had four or more children, from 2.1% in 2006 to 6.6% in 2019 (Table 1). 

Women residing in the South (2006 – 5.1%; 2013 – 4.5%; 2019 – 6.4%) and Southeast (2006 – 7.5%; 2013 – 5.7%; 2019 – 8.1%) reported higher prevalences of partner vasectomy in all years compared with those living in the North and Northeast (Table 1). This pattern was also observed for women living in urban areas (2013 – 4.5%; 2019 – 5.9%) compared with rural areas, and for those with private health insurance (2006 – 9.0%; 2013 – 6.2%; 2019 – 8.6%) compared with those without (Table 1). 

Women living with a partner and with higher education reported higher prevalences of partner vasectomy in 2006 (12.8%) and 2013 (6.7%) compared with those who attended elementary school. This was also observed for White women (8.4% in 2006; 6.6% in 2013) compared with Black and Brown women (4.8% in 2006; 4.0% in 2013) (Table 2). For the group of all women, this pattern was observed only in 2006 (Table 1). 

Women aged 35–39 years (2006 – 47.6%; 2013 – 32.5%; 2019 – 23.4%), Black and Brown (2006 – 37.1%; 2013 – 27.2%; 2019 – 20.6%), who attended elementary education (2006 – 43.4%; 2013 – 36.8%; 2019 – 30.8%), living in rural areas (2006 – 40.0%; 2013 – 33.0%; 2019 – 26.8%), without private health insurance (2006 – 36.3%; 2013 – 25.3%; 2019 – 20.0%), and who had three births (2006 – 64.2%; 2013 – 54.0%; 2019 – 44.1%) showed higher prevalences of tubal ligation (Table 3). This was also observed for those living with a partner (Table 4). 

**Table 4:** Prevalence (%) and 95% confidence interval (95%CI) of tubal ligation among Brazilian women of reproductive age living with a partner, according to sociodemographic characteristics. Brazil, 2006, 2013, and 2019

Sociodemographic characteristics	2006^a^	2013^b^	2019^c^
	%^d^ (95%CI)	%^d^ (95%CI)	%^d^ (95%CI)
Total	36.3 (34.5; 38.2)	26.7 (25.2; 28.3)	21.8 (20.6; 23.0)
**Age group** (years)			
15–19^and^	0.2 (0.0; 0.8)	-	0.2 (0.0; 0.08)
18–19^f^	-	0.2 (0.0; 1.2)	-
20–24	5.3 (3.7; 7.6)	3.3 (1.9; 5.6)	3.0 (1.9; 4.7)
25–29	20.5 (17.3; 24.1)	10.8 (8.9; 13.1)	10.8 (7.3; 15.8)
30–34	36.6 (32.8; 40.7)	22.5 (19.8; 25.4)	17.1 (15.0; 19.5)
35–39	47.6 (43.3; 51.9)	33.9 (30.7; 37.3)	24.8 (22.3; 27.6)
40–44	51.5 (45.7; 57.2)	43.8 (40.0; 47.7)	31.8 (28.7; 35.0)
45–49	67.9 (63.1; 72.3)	48.0 (42.7; 53.4)	39.5 (35.2; 43.9)
**Race/skin color**			
White	29.7 (27.1; 32.4)	20.3 (18.3; 22.3)	16.5 (14.7; 18.5)
Black/Brown	41.1 (38.7; 43.6)	32.4 (30.4; 34.5)	25.5 (23.8; 27.3)
**Education Level**			
Elementary School	44.5 (41.9; 47.2)	39.3 (36.7; 41.9)	34.3 (31.9; 36.8)
High School	25.6 (23.1; 28.3)	19.8 (17.9; 21.9)	18.6 (16.8; 20.6)
Higher Education	24.9 (20.4; 30.0)	16.1 (13.7; 18.9)	13.1 (11.2; 15.2)
Region			
Central-West	46.6 (43.4; 49.9)	36.6 (33.1; 40.2)	26.9 (23.4; 30.6)
North	52.6 (48.7; 56.7)	38.0 (34.3; 41.8)	35.0 (31.8; 38.4)
Northeast	46.5 (42.7; 50.4)	38.9 (36.2; 41.6)	31.6 (29.5; 33.8)
Southeast	30.0 (26.8; 33.4)	20.7 (18.0; 23.7)	16.1 (13.9; 18.5)
South	23.4 (20.2; 27.0)	10.4 (8.1; 13.2)	10.6 (8.6; 12.8)
**Place of residence**			
Urban Area	35.2 (33.1; 37.3)	25.2 (23.6; 26.9)	20.2 (18.9; 21.6)
Rural Area	41.6 (37.3; 46.0)	35.0 (31.7; 38.4)	30.7 (27.9; 33.7)
**Health Insurance**			
No	40.0 (37.9; 42.1)	30.2 (28.4; 32.0)	25.3 (23.8; 26.8)
Yes	26.7 (23.2; 30.4)	18.7 (16.2; 21.5)	13.2 (11.4; 15.3)
Parity			
None	1.5 (0.4; 5.1)	9.1 (3.0; 24.3)	6.8 (3.2; 14.0)
1	3.9 (2.6; 5.7)	1.8 (1.3; 2.4)	2.7 (2.0; 3.7)
2	42.3 (39.2; 45.4)	30.3 (27.9; 32.9)	25.0 (22.9; 27.2)
3	63.8 (59.5; 67.9)	56.3 (52.1; 60.5)	46.4 (42.0; 50.9)
4+	43.7 (39.5; 48.0)	29.1 (26.2; 32.2)	50.5 (45.8; 55.1)

^a^Results based on data from the 2006 Brazilian National Demographic and Health Survey of Children and Women (PNDS) (n=8,023); ^b^Results based on data from the 2013 National Health Survey (PNS) (n=9,994); ^c^Results based on data from the 2019 PNS (n=10,744); ^d^Population estimates; ^e^Age group used for analyses with data from the 2006 PNDS and 2019 PNS; ^f^Age group used with data from the 2013 PNS.

**Table 3:** Prevalence (%) and 95% confidence interval (95%CI) of tubal ligation among all Brazilian women of reproductive age, according to sociodemographic characteristics. Brazil, 2006, 2013, and 2019

Sociodemographic characteristics	2006^a^	2013^b^	2019^c^
	%^d^ (95%CI)	%^d^ (95%CI)	%^d^ (95%CI)
Total	32.2 (30.6; 33.9)	22.0 (20.8; 23.2)	17.3 (16.4; 18.2)
**Age group** (years)			
15–19^e^	0.0 (0.0; 3.5)	-	0.0 (0.0; 0.2)
18–19^f^	-	0.3 (0.0; 1.7)	-
20–24	3.5 (2.5; 5.0)	1.9 (1.2; 3.1)	1.5 (1.0; 2.3)
25–29	17.4 (14.8; 20.3)	8.4 (7.0; 10.0)	8.1 (5.7; 11.4)
30–34	34.0 (30.4; 37.8)	19.9 (17.7; 22.3)	15.3 (13.6; 17.2)
35–39	47.6 (43.6; 51.6)	32.5 (29.6; 35.5)	23.4 (21.2; 25.7)
40–44	51.7 (46.7; 56.6)	40.1 (36.9; 43.5)	30.0 (27.4; 32.7)
45–49	68.0 (63.7; 72.1)	47.1 (42.4; 52.0)	37.1 (33.4; 40.9)
**Race/skin color**			
White	26.2 (24.0; 28.6)	16.1 (14.7; 17.7)	12.9 (11.6; 14.3)
Black/Brown	37.1 (34.9; 39.3)	27.2 (25.6; 28.9)	20.6 (19.3; 21.9)
**Education Level**			
Elementary School	43.4 (41.0; 45.9)	36.8 (34.5; 39.0)	30.8 (29.7; 23.9)
High School	21.2 (19.3; 23.3)	15.5 (14.0; 17.0)	14.8 (13.5; 16.1)
Higher Education	18.1 (15.0; 21.7)	11.7 (10.0; 13.6)	9.2 (8.0; 10.5)
Region			
Central-West	43.4 (40.4; 46.4)	30.1 (27.2; 33.1)	22.8 (20.1; 25.6)
North	47.8 (44.2; 51.4)	31.9 (28.8; 35.1)	27.9 (25.3; 30.5)
Northeast	43.9 (40.4; 47.5)	33.8 (31.6; 36.1)	25.7 (24.1; 27.3)
Southeast	25.2 (22.5; 28.1)	15.8 (13.9; 18.0)	12.1 (10.6; 13.7)
South	20.9 (18.3; 23.8)	9.1 (7.3; 11.4)	8.7 (7.3; 10.4)
**Place of residence**			
Urban Area	30.7 (29.0; 32.6)	20.4 (19.2; 21.7)	16.0 (15.0; 17.0)
Rural Area	40.0 (35.6; 44.5)	33.0 (29.8; 36.4)	26.8 (24.4; 29.3)
**Health Insurance**			
No	36.3 (34.5; 38.2)	25.3 (23.9; 26.7)	20.0 (19.0; 21.1)
Yes	21.9 (19.1; 25.0)	14.6 (12.7; 16.7)	10.2 (8.9; 11.7)
Parity			
None	1.0 (0.3; 3.5)	4.6 (1.7; 11.6)	3.5 (1.8; 6.8)
1	4.0 (2.9; 5.5)	1.7 (1.3; 2.3)	2.6 (2.0; 3.3)
2	43.8 (40.8; 46.9)	29.9 (27.6; 32.3)	24.1 (22.2; 26.0)
3	64.2 (60.2; 68.1)	54.0 (50.1; 57.8)	44.1 (40.3; 48.0)
4+	27.4 (24.4; 30.6)	17.7 (16.0; 19.5)	48.0 (44.0; 51.9)

^a^Results based on data from the 2006 Brazilian National Demographic and Health Survey of Children and Women (PNDS) (n=11,324); ^b^Results based on data from the 2013 National Health Survey (PNS) (n=14,394); ^c^Results based on data from the 2019 PNS (n=16,640); ^d^Population estimates; ^e^Age group used for analyses with data from the 2006 PNDS and 2019 PNS; ^f^Age group used with data from the 2013 PNS.

Women residing in the South (2006 – 20.9%; 2013 – 9.1%; 2019 – 8.7%) and Southeast (2006 – 25.2%; 2013 – 15.8%; 2019 – 12.1%) reported lower prevalences of tubal ligation (Tables 3 and 4). A reduction in tubal ligation prevalence according to these characteristics was observed between 2006 and 2019, except for women who had four or more births, which showed a 75.2% increase among all women (Table 3). This was not observed for the group of women living with a partner (Table 4).

## Discussion 

These findings revealed an increase in partner-reported vasectomy and a decrease in tubal ligation between 2006 and 2019, although the prevalence of surgical methods among men remained lower than among women. Women with greater social vulnerability, such as Black and Brown women, those with a lower education level, living in rural areas, and without private health insurance, showed higher prevalences of tubal ligation. Women with more favorable sociodemographic conditions, such as higher education level, living in urban areas, residing in the South and Southeast regions, and having access to private health insurance, reported higher prevalences of partners who had undergone vasectomy. These results highlighted the persistence of social inequalities in access to permanent contraceptive methods.

As limitations of this study, vasectomy was reported by women regarding their partners, which may reflect a gender bias inherent in surveys on this topic. Data from the 1986 demographic survey and the 1996 PNDS were not included due to a lack of sample comparability. If included, a continuous increase in vasectomies would have been observed, similar to that seen in 2006, 2013, and 2019 (7,8). For tubal ligation, an increase would have been observed between 1986 and 1996, followed by a decrease during the studied period (7,8). 

Another limitation referred to the comparability of the age and education variables. However, for the latter, equivalence between education level classification and years of schooling was considered. It was also not possible to perform a trend analysis due to the limited number of time points; however, observing the evolution of these indicators over time emphasizes the need for ongoing monitoring to inform public policies in sexual and reproductive health.

In France, between 2010 and 2022, vasectomy increased, with higher use among men aged 41–44 years and those with better socioeconomic conditions (21), corroborating this finding. For tubal ligation, an increase was observed between 2010 and 2013, followed by a decrease between 2013 and 2022 among women with less favorable socioeconomic conditions (21). This scenario shows that access to permanent contraceptive methods also presents inequities in developed countries. However, in France, between 2021 and 2022, more vasectomies than tubal ligations were performed (21), a situation distinct from that in Brazil. 

The Women’s Empowerment Index (WEI) is a measure based on ten indicators, including access to modern contraceptive methods, such as long-acting methods. According to this index, Brazil presented medium-low empowerment (0.637), behind the United States (0.752) and the United Kingdom (0.778), both with medium-high empowerment (22). It can be observed that the empowerment of Brazilian women is still under development, which is reflected in access to long-acting contraceptive methods, used by only 5% of Brazilian women (12,13). 

This fact may explain the choice of tubal ligation among women who have already reached their desired number of children due to its high effectiveness. However, the importance of professional counseling that promotes free and informed choice should be emphasized, since the desire to have more children may change over time, and tubal ligation reversal is not widely accessible and has a low success rate (1).

The Gender Inequality Index (GII) is composed of indicators related to reproductive health, empowerment, and labor market participation, and is associated with the use of permanent contraceptive methods. For example, the prevalence of vasectomy in 2019 was 14% in the United States and 5% in Brazil, while the prevalence of tubal ligation was 19.0% and 21.8%, respectively. According to the Gender Inequality Index (GII), the ranking of these countries is 12th (United States) and 31st (Brazil) (23). The higher the Gender Inequality Index (GII), the less evident the male role in reproductive planning. 

Globally, women use contraceptive methods more than men, reinforcing gender inequality in the field of contraception. In 2019 in Brazil, the prevalence of male condom use was 20%, whereas oral contraceptive use was 40% (12). This disparity is exacerbated by the greater availability of contraceptives for women (female condom, diaphragm, intrauterine device, pills, injectables, implants), while men have only the male condom and vasectomy. 

Male methods have been investigated, but clinical trials were discontinued due to side effects such as depression and changes in libido (24). Other research has focused on oral and topical methods to increase ease of use (24). If proven effective and well-tolerated, these methods have the potential for clinical use. However, barriers to the development of hormonal male contraceptives persist, including a lack of investment, concerns about side effects, spermatogenic rebound, unproven reversibility, and the effectiveness of non-hormonal methods (25). These challenges reinforce the persistence of gender inequalities, with women continuing to be the main actors in reproductive planning. 

Ministry of Health policies focused on men’s health (26,27) have aimed, among other objectives, to promote vasectomy through the implementation and expansion of the Men’s Health Care system (27). This may have contributed to the increase in vasectomy prevalence observed during the study period. Other possible explanations for this finding include: greater awareness among men regarding the safety and effectiveness of vasectomy; increased male participation in contraceptive decision-making; a higher proportion of couples wishing to have fewer or no children, or who have already achieved their desired number of children; economic difficulties in raising children; and greater knowledge among partners that tubal ligation carries higher risk of complications than vasectomy (28).

Hypotheses for the low prevalence of vasectomy in Brazil include persistent patriarchal beliefs that contraception is the woman’s responsibility; lack of knowledge about this contraceptive method; low demand by men for health services; and prejudice or fear related to the mistaken belief that vasectomy causes impotence (23,29). This context of limited information may perpetuate gender inequality and contribute to the sociodemographic disparities observed in the prevalence of these methods.

Sociodemographic inequalities may be related to the use of each surgical method by different groups. Higher prevalences of tubal ligation were observed among women with poorer sociodemographic conditions, such as lower education level, Black and Brown women, those without private health insurance, living in rural areas, and who had higher parity. In contrast, women with more favorable conditions, such as residing in urban areas and in the South and Southeast regions, having private health insurance, and a higher education level, reported higher prevalences of partner vasectomy. 

This study reinforced existing inequalities in the type of contraceptive used by Brazilian women, as shown in previous studies (12,13). However, it advanced knowledge by highlighting this difference between tubal ligation and vasectomy, pointing to the need to expand access to vasectomy among the socially vulnerable population.

Women with greater social vulnerability face more obstacles in obtaining information about contraceptive methods and how to access them, which may result in lower decision-making power regarding which method to use. Education level is one of the main determinants of contraceptive use, as it provides greater access to information and knowledge, and constitutes a vehicle for socioeconomic mobility and sexual and reproductive autonomy (30), which helps explain the observed inequalities. 

Sexual and reproductive autonomy refers to reproductive rights, the choice of timing for pregnancy, the desired number of children, and decision-making regarding contraceptive use (31). Considering the inequities in access to contraceptive methods faced by these women, it can be concluded that they are being deprived of full autonomy, highlighting the need to advance equitable access to reproductive planning in the country.

In 2019, approximately half of Brazilian women used hormonal methods (12). Only in 2013 did tubal ligation cease to be the most commonly used contraceptive method among Brazilian women (13). This reality reaffirms that the choice of method is related to the information women receive and their opportunities for access. 

Long-acting contraceptive methods, which are more effective, are more commonly used by women with higher income, education, and private health insurance (12), as observed for vasectomy, reinforcing the importance of greater access to contraceptive methods and information to achieve contraceptive justice. Therefore, the care provided to the population regarding contraception should include information on indications, how to use the method, and adverse effects, advantages, and disadvantages. Regular clinical follow-up should also be included, ensuring quality of life, health, and reproductive dignity, as well as greater autonomy in choosing a contraceptive method (32). 

The findings of this study, showing an increase in vasectomy prevalence and a reduction in tubal ligation between 2006 and 2019, represent progress in sexual and reproductive health in the Brazilian population, resulting from greater access to alternative contraceptive options. On the other hand, gender and sociodemographic inequalities in access to permanent methods persist in the country. 

Setbacks in public policies aimed at ensuring male and female surgical sterilization within SUS, under the scope of Sexual and Reproductive Health (13), such as the growing conservatism in Brazilian society (33), may further hinder male participation in contraception. It is recognized that results in future population surveys may differ, given that elective surgeries were interrupted during the COVID-19 pandemic (34). This may have influenced the reduced use of permanent methods, despite legislative changes that facilitated access, including the removal of spousal authorization—a step forward in the fight for gender equality. 

Future studies will be necessary to continue monitoring this temporal evolution. Continuous monitoring of contraceptive use indicators in the country could more effectively guide investments in qualified and equitable access, considering the needs of each individual and respecting the exercise of sexual and reproductive rights.
