# Supplementary material for: Prevalence of vasectomy and tubal ligation: time series analysis Brazil, 2006–2019
Source: Epidemiol Serv Saude. 2025 Dec 19;35:e20240474. doi: 10.1590/S2237-96222026v35e20240474.en (PMC12716122; doi:10.1590/S2237-96222026v35e20240474.en)
Supplement: Translation

# Translation: Prevalência de vasectomia e laqueadura tubária: análise de série temporal, Brasil, 2006-2019

**DOI:** 10.1590/S2237-96222026v35e20240474.pt

**Authors:** Patrícia Fernandes Murta, Fernanda Gontijo Araújo, Torcata Amorim, Gisele Nepomuceno de Andrade, Mariana Santos Felisbino-Mendes

**Affiliations:** 1 Universidade Federal de Minas Gerais, Belo Horizonte, MG, Brasil

**Keywords:** Vasectomia, Esterilização Tubária, Planejamento Familiar, Desigualdades em Saúde, Inquéritos Epidemiológicos

## Abstract

**Objetivo:**

Analisar a evolução temporal da prevalência de vasectomia e laqueadura tubária em 2006, 2013 e 2019 conforme características sociodemográficas da população brasileira em idade reprodutiva.

**Métodos:**

Análise de série temporal que utilizou dados secundários de três inquéritos nacionais comparáveis, os quais investigaram o planejamento reprodutivo das mulheres brasileiras. Estimou-se prevalência de vasectomia do parceiro referida pelas mulheres e laqueadura com intervalos de confiança de 95%.

**Resultados:**

Houve aumento da prevalência de vasectomia dos parceiros de 4,2% para 5,6% para todas as mulheres e 5,2% para 7,8% para as que moram com o companheiro entre 2013 e 2019. Houve redução da laqueadura de 32,2% para 17,3% para todas as mulheres e de 36,3% para 21,8% para as que moram com o companheiro entre 2006 e 2019. Mulheres pretas e pardas (2006 – 37,1%; 2013 – 27,2%; 2019 – 20,6%), com ensino fundamental (2006 – 43,4%; 2013 – 36,8%; 2019 – 30,8%), que vivem nas áreas rurais (2006 – 40,0%; 2013 – 33,0%; 2019 – 26,8%) e que tiveram três partos (2006 – 64,2%; 2013 – 54,0%; 2019 – 44,1%) apresentaram maiores prevalências de laqueadura em todo o período analisado. Mulheres que tinham plano de saúde (2006 – 9,0%; 2013 – 6,2%; 2019 – 8,6%) relataram maiores prevalências de vasectomia em seus parceiros.

**Conclusão:**

Apesar dos avanços observados pelo aumento da prevalência da vasectomia e pela redução da laqueadura, permanecem desigualdades de gênero e sociais quanto ao uso desses métodos, o que apontou a necessidade de ampliar o acesso aos métodos contraceptivos de longa duração de forma equitativa no país.

**Aspectos éticos**

Esta pesquisa utilizou bancos de dados de domínio público e anonimizados.

## Introdução 

A vasectomia e a laqueadura tubária, métodos contraceptivos permanentes, são procedimentos cirúrgicos invasivos utilizados por indivíduos e casais que não desejam ter filhos ou que já atingiram o número de filhos almejados (1). Enquanto a laqueadura tubária é um dos métodos mais utilizados no mundo, com cerca de 19% de usuárias, a vasectomia é utilizada por menos de 3% dos homens (1).

No Brasil, na década de 1980, houve popularização da laqueadura tubária, realizada principalmente em mulheres de baixa renda e regiões mais pobres (2). Em 1983, o Programa de Assistência Integral à Saúde da Mulher propôs ações para promoção e prevenção de saúde da mulher e para o planejamento reprodutivo (3). Entretanto, tinham ênfase na laqueadura tubária como método contraceptivo de alta eficácia, realizada durante a cesárea (4), para cobrir seus custos, pois não era reembolsável pelo sistema público de saúde (5). 

Esse método contraceptivo passou a figurar como principal escolha de contracepção entre os casais (6), o que resultou em aumento de mulheres em união esterilizadas, de 27% em 1986 para 40% em 1996 (7,8). Em 1986, 75% das esterilizações realizadas no país foram feitas durante a cesárea (9). A laqueadura tubária também era obtida de forma gratuita como favor político, principalmente na região Nordeste (5). 

Em 1992, a Comissão Parlamentar de Inquérito investigou essa realidade no país e divulgou relatório, no qual apontava a demanda crescente por contracepção e escassas alternativas de métodos contraceptivos, principalmente para a população de baixa renda (6). Com isso, em 1996 foi promulgada a Lei do Planejamento Familiar. Essa política pública visava regulamentar a realização da esterilização cirúrgica e aumentar o acesso a outros métodos contraceptivos no âmbito do Sistema Único de Saúde (SUS) (10). Somente em 2022, essa lei foi revisada, e, a partir de então, homens e mulheres com idade acima de 21 anos podem recorrer à esterilização cirúrgica, inclusive durante o parto (11).

Os métodos contraceptivos permanentes são importantes para as mulheres, em contexto no qual o acesso a outros métodos com eficácia aproximada, como o dispositivo intrauterino, ainda não tem ampla e consolidada oferta. No Brasil, os contraceptivos de longa duração são mais usados por mulheres com plano de saúde, maior renda e escolaridade, enquanto os métodos permanentes são mais utilizados pelas mulheres com baixa escolaridade (12). Tal realidade revela iniquidades em relação ao uso de métodos contraceptivos no Brasil (12,13). 

A maioria dos estudos trata do conjunto das esterilizações ou se restringe à laqueadura tubária (5,6,9,12), não permitindo acompanhar o padrão de cada tipo de esterilização no país. Também restringe a avaliação do uso desses métodos às mulheres em união (5,6,9), fazendo-se necessário avaliar separadamente a evolução temporal do uso desses métodos contraceptivos no Brasil. 

Este estudo visou analisar a evolução temporal da prevalência de vasectomia e laqueadura tubária no Brasil em 2006, 2013 e 2019, de acordo com características sociodemográficas da população em idade reprodutiva. 

## Métodos 

### Delineamento do estudo e contexto

Trata-se de estudo epidemiológico, de série temporal, com dados secundários de mulheres brasileiras em idade reprodutiva que responderam questões sobre planejamento reprodutivo em inquéritos populacionais de saúde no país.

### População de estudo

Para esta análise, consideraram-se as mulheres brasileiras em idade reprodutiva, usuárias de métodos contraceptivos e que não estavam grávidas. Essa população foi subdividida em dois grupos: todas as mulheres e mulheres que moravam com o companheiro. Esse procedimento foi realizado nos três inquéritos.

### Fonte de dados e tamanho do estudo

Foram utilizados três inquéritos populacionais: a Pesquisa Nacional de Demografia e Saúde da Criança e da Mulher (PNDS) 2006, a Pesquisa Nacional de Saúde (PNS) 2013 e a PNS 2019.

A PNDS 2006 foi uma pesquisa nacional domiciliar realizada com amostragem probabilística complexa em dois estágios. O primeiro estágio compreendeu as unidades primárias de amostragem representadas pelos setores censitários. O segundo estágio consistiu nas unidades secundárias de amostragem, representadas pelas unidades domiciliares. A amostra incluiu domicílios particulares e comunidades, selecionados em 10 estratos para as cinco macrorregiões do país (Norte, Nordeste, Sul, Sudeste e Centro-Oeste), e abrangeu áreas urbanas e rurais. Foram selecionados 14.617 domicílios e 15.575 mulheres entre 15 e 49 anos foram entrevistadas (14,15). 

A PNS, inquérito nacional domiciliar, foi realizada em 2013 e 2019, cujas amostras corresponderam a uma subamostra da Amostra Mestra do Sistema Integrado de Pesquisas Domiciliares do Instituto Brasileiro de Geografia e Estatística. Amostra Mestra consistiu em um conjunto de unidades primárias de amostragem de áreas selecionadas para atender a pesquisas do Sistema Integrado de Pesquisas Domiciliares, sendo estas unidades primárias de amostragem utilizadas no planejamento amostral da PNS. 

Amostra da PNS 2013 foi elaborada a partir de três estágios: seleção da subamostra das unidades primárias de amostragem em cada estrato da Amostra Mestra com probabilidade proporcional ao tamanho; seleção por amostragem aleatória simples de domicílios em cada unidade primária de amostragem selecionada; e seleção por amostragem aleatória simples da pessoa acima de 18 anos de idade entre todos os moradores adultos do domicílio (16). Foram selecionados 63.348 domicílios, e 22.621 mulheres entre 18 e 49 anos foram entrevistadas em 2013 (13,17). 

Amostra da PNS 2019 também seguiu a mesma metodologia, porém os entrevistados deveriam ter 15 anos ou mais de idade (18). Foram selecionados 94.114 domicílios, e 27.249 mulheres em idade reprodutiva foram entrevistadas (12,19). 

### Variáveis 

O desfecho foi o uso de métodos contraceptivos permanentes referido pelas mulheres em idade reprodutiva, os quais incluíram a vasectomia pelos parceiros e a laqueadura tubária pela mulher. 

As variáveis explicativas foram as características sociodemográficas das mulheres. A faixa etária foi categorizada de 15-19 anos para a PNDS 2006 e a PNS 2019 e 18-19 anos para a PNS 2013. As outras categorias de faixa etária foram iguais para os três inquéritos: 20-24, 25-29, 30-34, 40-44 e 45-49 anos. As demais variáveis foram categorizadas da seguinte forma: raça/cor da pele (branca, preta/parda); escolaridade (ensino fundamental, ensino médio, ensino superior); região de moradia (Centro-Oeste, Norte, Nordeste, Sudeste, Sul); local da residência (urbana, rural); posse de plano de saúde (não, sim); e número de partos (nenhum, um, dois, três, quatro ou mais). Foram excluídas as categorias amarela e indígena, devido ao número amostral muito pequeno, não garantindo representatividade desses grupos populacionais. 

### Viés 

A vasectomia do parceiro referida pelas mulheres poderia repercutir em menores prevalências do uso deste método, uma vez que as mulheres poderiam não saber se os parceiros fizeram essa cirurgia, principalmente mulheres solteiras. As diferenças nas faixas etárias também poderiam dificultar a comparabilidade, principalmente o fato de a PNS em 2013 não ter incluído as mulheres de 15-17 anos no desenho amostral.

### Análise estatística

Estimou-se a prevalência de vasectomia do parceiro referida pelas mulheres e da laqueadura tubária, considerando no numerador as usuárias desses métodos e no denominador todas as mulheres que faziam uso de algum método contraceptivo. Em seguida, a prevalência do uso de cada método contraceptivo permanente foi estimada segundo as características sociodemográficas. Todas as estimativas foram obtidas com intervalos de confiança de 95% (IC95%) para analisar diferenças entre os grupos. 

Os dados foram analisados utilizando o programa estatístico Stata, no módulo survey, a fim de obter estimativas populacionais, considerando estrato, conglomerado e peso do indivíduo. Os pesos da base da PNS 2013 foram reponderados, o que permitiu a comparação entre 2013 e 2019 (20).

### Resultados 

Observou-se aumento da prevalência de vasectomia do parceiro referida pelas mulheres brasileiras em idade reprodutiva entre 2013 e 2019, de 4,2% para 5,6% (Tabela 1) – acréscimo de 33,3%, ainda maior no grupo de mulheres que moravam com companheiro, de 5,2% para 7,8% (Tabela 2; Figura 1A). A prevalência de laqueadura tubária foi 32,2% em 2006 e 17,3% em 2019 (redução de 46,3%). Para as mulheres que moravam com companheiro, essa redução foi 39,9%, variando de 36,3% em 2006 para 21,8% em 2019 (Figura 1B).

**Tabela 2:** Prevalência (%) e intervalo de confiança de 95% (IC95%) de vasectomia dos parceiros referida por mulheres brasileiras em idade reprodutiva e que moram com o companheiro, de acordo com as características sociodemográficas. Brasil, 2006, 2013 e 2019

Características sociodemográficas	2006^a^	2013^b^	2019^c^
	%^d^ (IC95%)	%^d^ (IC95%)	%^d^ (IC95%)
Total	6,3 (5,4; 7,4)	5,2 (4,5; 6,1)	7,8 (7,0; 8,8)
**Faixa etária** (anos)			
15-19^e^	0,9 (0,2; 3,3)	-	0,3 (0,0; 2,2)
18-19^f^	-	0,0 (0,0; 0,7)	-
20-24	0,5 (0,2; 1,4)	0,4 (0,1; 1,1)	0,4 (0,2; 1,1)
25-29	4,2 (2,4; 7,3)	2,9 (1,7; 4,8)	2,6 (1,6; 4,2)
30-34	8,8 (6,3; 12,1)	5,4 (4,0; 7,3)	4,7 (3,4; 6,6)
35-39	7,5 (5,4; 10,4)	5,7 (4,3; 7,6)	11,4 (9,4; 13,7)
40-44	10,0 (7,4; 13,5)	10,0 (7,7; 12,9)	11,6 (9,3; 14,4)
45-49	6,8 (4,7; 9,8)	6,4 (4,3; 9,4)	13,6 (10,6; 17,3)
**Raça/cor da pele**			
Branca	8,4 (6,8; 10,2)	6,6 (5,4; 8,1)	9,0 (7,7; 10,6)
Preta/parda	4,8 (3,7; 6,2)	4,0 (3,2; 5,1)	7,0 (6,0; 8,2)
Escolaridade			
Ensino fundamental	4,9 (3,8; 6,3)	3,8 (2,8; 5,0)	6,5 (5,0; 8,4)
Ensino médio	6,5 (5,0; 8,4)	6,0 (4,8; 7,4)	7,7 (6,5; 9,2)
Ensino superior	12,8 (9,5; 17,1)	6,7 (5,1; 8,7)	9,6 (8,0; 11,5)
Região			
Centro-Oeste	4,7 (3,4; 6,2)	5,8 (4,3; 7,8)	7,7 (5,8; 10,1)
Norte	0,9 (0,5; 1,8)	1,4 (0,7; 2,5)	1,8 (1,3; 2,7)
Nordeste	1,8 (1,1; 2,9)	2,5 (1,6; 3,8)	2,6 (2,0; 3,5)
Sudeste	10,3 (8,3; 12,7)	7,8 (6,2; 9,6)	12,1 (10,3; 14,2)
Sul	6,6 (4,9; 8,7)	5,4 (4,0; 7,2)	8,7 (6,9; 10,8)
**Local da residência**			
Urbana	7,2 (6,1; 8,4)	5,9 (5,0; 6,8)	8,7 (7,7; 9,7)
Rural	2,5 (1,1; 5,9)	1,8 (1,1; 3,0)	3,2 (2,2; 4,5)
**Posse de plano de saúde**			
Não	4,2 (3,3; 5,2)	4,0 (3,2; 4,8)	6,1 (5,2; 7,2)
Sim	12,1 (9,7; 15,0)	8,2 (6,7; 10,1)	12,0 (10,2; 14,1)
**Número de partos**			
Nenhum	6,0 (1,1; 26,4)	0,4 (0,0; 3,0)	5,9 (1,5; 19,8)
1	2,7 (1,8; 4,1)	2,2 (1,4; 3,4)	4,0 (3,0; 5,4)
2	9,9 (8,1; 12,0)	8,0 (6,5; 9,7)	12,4 (10,7; 14,3)
3	7,2 (4,9; 10,5)	6,7 (4,8; 9,2)	9,6 (7,3; 12,5)
4+	4,0 (2,4; 6,5)	4,1 (2,9; 6,0)	8,1 (5,1; 12,5)

^a^Resultados com dados da Pesquisa Nacional de Demografia e Saúde da Criança e da Mulher (PNDS) 2006 (n=8.023); ^b^Resultados com dados da Pesquisa Nacional de Saúde (PNS) 2013 (n=9.994); ^c^Resultados com dados da PNS 2019 (n=10.744); ^d^Estimativas populacionais; ^e^Faixa etária utilizada para análise com dados da PNDS 2006 e da PNS 2019; ^f^Faixa etária utilizada com dados da PNS 2013.

**Tabela 1:** Prevalência (%) e intervalo de confiança de 95% (IC95%) de vasectomia dos parceiros referida por todas as mulheres brasileiras em idade reprodutiva, de acordo com as características sociodemográficas. Brasil, 2006, 2013 e 2019

Características sociodemográficas	2006^a^	2013^b^	2019^c^
	%^d^ (IC95%)	%^d^ (IC95%)	%^d^ (IC95%)
Total	4,9 (4,1; 5,7)	4,2 (3,6; 4,8)	5,6 (5,0; 6,2)
**Faixa etária** (anos)			
15-19^e^	0,4 (0,0; 1,5)	-	0,4 (0,1; 1,1)
18-19^f^	-	0,0 (0,0; 1,0)	-
20-24	0,4 (0,2; 0,9)	0,3 (0,1; 0,7)	0,2 (0,1; 0,5)
25-29	3,2 (1,9; 5,5)	2,3 (1,4; 3,7)	2,0 (1,3; 3,1)
30-34	7,3 (5,3; 10,1)	4,4 (3,3; 5,9)	3,8 (2,7; 5,2)
35-39	7,0 (5,2; 9,5)	5,1 (3,8; 6,8)	9,1 (7,6; 10,9)
40-44	8,5 (6,2; 11,4)	9,0 (7,0; 11,5)	9,5 (7,7; 11,7)
45-49	5,4 (3,7; 7,8)	6,4 (4,5; 9,1)	11,8 (9,3; 14,8)
**Raça/cor da pele**			
Branca	6,4 (5,3; 7,8)	5,2 (4,3; 6,3)	6,3 (5,4; 7,4)
Preta/parda	3,7 (2,9; 4,9)	3,2 (2,6; 4,0)	5,1 (4,4; 5,9)
Escolaridade			
Ensino fundamental	4,1 (3,2; 5,3)	3,5 (2,7; 4,5)	5,3 (4,1; 6,7)
Ensino médio	4,6 (5,6; 5,9)	4,5 (3,7; 5,6)	5,3 (4,5; 6,3)
Ensino superior	8,0 (6,0; 10,7)	4,6 (3,5; 6,0)	6,2 (5,2; 7,4)
Região			
Centro-Oeste	3,9 (3,0; 5,3)	4,6 (3,5; 6,1)	5,8 (4,5; 7,5)
Norte	0,8 (0,4; 1,6)	1,2 (0,7; 2,0)	1,8 (1,2; 2,5)
Nordeste	1,5 (0,9; 2,4)	2,2 (1,4; 3,5)	2,1 (1,6; 2,6)
Sudeste	7,5 (6,1; 9,2)	5,7 (4,7; 7,0)	8,1 (6,9; 9,5)
Sul	5,1 (3,8; 6,8)	4,5 (0,3; 6,0)	6,4 (5,2; 8,0)
**Local da residência**			
Urbana	5,3 (4,6; 6,3)	4,5 (3,9; 5,2)	5,9 (5,3; 6,7)
Rural	2,2 (0,9; 5,0)	1,8 (1,1; 2,8)	2,9 (2,1; 4,0)
**Posse de plano de saúde**			
Não	3,2 (2,6; 4,0)	3,2 (2,7; 3,9)	4,4 (3,8; 5,1)
Sim	9,0 (7,2; 11,2)	6,2 (5,1; 7,6)	8,6 (7,3; 10,1)
**Número de partos**			
Nenhum	4,9 (1,2; 18,5)	0,5 (0,1; 1,8)	3,4 (1,2; 9,6)
1	2,4 (1,6; 3,5)	1,9 (1,3; 2,9)	3,3 (2,5; 4,3)
2	9,0 (7,4; 10,9)	7,0 (5,7; 8,6)	10,5 (9,1; 12,1)
3	6,2 (4,2; 9,1)	6,3 (4,6; 8,6)	8,4 (6,5; 10,7)
4+	2,1 (1,2; 3,5)	2,8 (2,0; 3,8)	6,6 (4,3; 10,1)

^a^Resultados com dados da Pesquisa Nacional de Demografia e Saúde da Criança e da Mulher (PNDS) 2006 (n=11.324); ^b^Resultados com dados da Pesquisa Nacional de Saúde (PNS) 2013 (n=14.394); ^c^Resultados com dados da PNS 2019 (n=16.640); ^d^Estimativas populacionais; ^e^Faixa etária utilizada para análise com dados da PNDS 2006 e da PNS 2019; ^f^Faixa etária utilizada com dados da PNS 2013.

**Figura 1:** Evolução temporal da prevalência: (A) vasectomia dos parceiros referida pelas mulheres; (B) laqueadura tubária. Brasil, 2006-2019

Houve aumento da prevalência de vasectomia do parceiro referida por todas as mulheres na faixa etária acima de 45 anos, de 5,4% em 2006 para 11,8% em 2019 (Tabela 1). Isso também foi observado para mulheres que moravam com companheiro (6,8% para 13,6%) (Tabela 2). Ocorreu aumento da prevalência de vasectomia do parceiro referida por todas as mulheres para àquelas que tiveram quatro ou mais filhos, de 2,1% em 2006 para 6,6% em 2019 (Tabela 1). 

As mulheres que residiam nas regiões Sul (2006 – 5,1%; 2013 – 4,5%; 2019 – 6,4%) e Sudeste (2006 – 7,5%; 2013 – 5,7%; 2019 – 8,1%) referiram maiores prevalências de vasectomia do parceiro em todos os anos avaliados quando comparadas às que viviam nas regiões Norte e Nordeste (Tabela 1). Isso também foi observado para mulheres que residiam em áreas urbanas (2013 – 4,5%; 2019 – 5,9%) em relação às que viviam em áreas rurais e que tinham plano de saúde (2006 – 9,0%; 2013 – 6,2%; 2019 – 8,6%) quando comparadas às que não tinham (Tabela 1). 

Mulheres que moravam com companheiro e tinham ensino superior referiram maior prevalência de vasectomia do parceiro em 2006 (12,8%) e 2013 (6,7%) em relação àquelas com ensino fundamental. Isso também foi observado para as brancas (8,4% em 2006; 6,6% em 2013) quando comparadas às pretas e pardas (4,8% em 2006; 4,0% em 2013) (Tabela 2). Para o grupo de todas as mulheres, esse padrão foi observado apenas em 2006 (Tabela 1). 

Mulheres na faixa etária 35-39 anos (2006 – 47,6%; 2013 – 32,5%; 2019 – 23,4%), pretas e pardas (2006 – 37,1%; 2013 – 27,2%; 2019 – 20,6%), com ensino fundamental (2006 – 43,4%; 2013 – 36,8%; 2019 – 30,8%), que residiam em áreas rurais (2006 – 40,0%; 2013 – 33,0%; 2019 – 26,8%), sem plano de saúde (2006 – 36,3%; 2013 – 25,3%; 2019 – 20,0%) e que tiveram três partos (2006 – 64,2%; 2013 – 54,0%; 2019 – 44,1%) apresentaram prevalências mais altas de laqueadura (Tabela 3). Isso também foi observado para aquelas que moravam com companheiro (Tabela 4). 

**Tabela 4:** Prevalência (%) e intervalo de confiança de 95% (IC95%) de laqueadura tubária em mulheres brasileiras em idade reprodutiva e que moram com o companheiro, de acordo com as características sociodemográficas. Brasil, 2006, 2013 e 2019

Características sociodemográficas	2006^a^	2013^b^	2019^c^
	%^d^ (IC95%)	%^d^ (IC95%)	%^d^ (IC95%)
Total	36,3 (34,5; 38,2)	26,7 (25,2; 28,3)	21,8 (20,6; 23,0)
**Faixa etária** (anos)			
15-19^e^	0,2 (0,0; 0,8)	-	0,2 (0,0; 0,08)
18-19^f^	-	0,2 (0,0; 1,2)	-
20-24	5,3 (3,7; 7,6)	3,3 (1,9; 5,6)	3,0 (1,9; 4,7)
25-29	20,5 (17,3; 24,1)	10,8 (8,9; 13,1)	10,8 (7,3; 15,8)
30-34	36,6 (32,8; 40,7)	22,5 (19,8; 25,4)	17,1 (15,0; 19,5)
35-39	47,6 (43,3; 51,9)	33,9 (30,7; 37,3)	24,8 (22,3; 27,6)
40-44	51,5 (45,7; 57,2)	43,8 (40,0; 47,7)	31,8 (28,7; 35,0)
45-49	67,9 (63,1; 72,3)	48,0 (42,7; 53,4)	39,5 (35,2; 43,9)
**Raça/cor da pele**			
Branca	29,7 (27,1; 32,4)	20,3 (18,3; 22,3)	16,5 (14,7; 18,5)
Preta/parda	41,1 (38,7; 43,6)	32,4 (30,4; 34,5)	25,5 (23,8; 27,3)
Escolaridade			
Ensino fundamental	44,5 (41,9; 47,2)	39,3 (36,7; 41,9)	34,3 (31,9; 36,8)
Ensino médio	25,6 (23,1; 28,3)	19,8 (17,9; 21,9)	18,6 (16,8; 20,6)
Ensino superior	24,9 (20,4; 30,0)	16,1 (13,7; 18,9)	13,1 (11,2; 15,2)
Região			
Centro-Oeste	46,6 (43,4; 49,9)	36,6 (33,1; 40,2)	26,9 (23,4; 30,6)
Norte	52,6 (48,7; 56,7)	38,0 (34,3; 41,8)	35,0 (31,8; 38,4)
Nordeste	46,5 (42,7; 50,4)	38,9 (36,2; 41,6)	31,6 (29,5; 33,8)
Sudeste	30,0 (26,8; 33,4)	20,7 (18,0; 23,7)	16,1 (13,9; 18,5)
Sul	23,4 (20,2; 27,0)	10,4 (8,1; 13,2)	10,6 (8,6; 12,8)
**Local da residência**			
Urbana	35,2 (33,1; 37,3)	25,2 (23,6; 26,9)	20,2 (18,9; 21,6)
Rural	41,6 (37,3; 46,0)	35,0 (31,7; 38,4)	30,7 (27,9; 33,7)
**Posse de plano de saúde**			
Não	40,0 (37,9; 42,1)	30,2 (28,4; 32,0)	25,3 (23,8; 26,8)
Sim	26,7 (23,2; 30,4)	18,7 (16,2; 21,5)	13,2 (11,4; 15,3)
**Número de partos**			
Nenhum	1,5 (0,4; 5,1)	9,1 (3,0; 24,3)	6,8 (3,2; 14,0)
1	3,9 (2,6; 5,7)	1,8 (1,3; 2,4)	2,7 (2,0; 3,7)
2	42,3 (39,2; 45,4)	30,3 (27,9; 32,9)	25,0 (22,9; 27,2)
3	63,8 (59,5; 67,9)	56,3 (52,1; 60,5)	46,4 (42,0; 50,9)
4+	43,7 (39,5; 48,0)	29,1 (26,2; 32,2)	50,5 (45,8; 55,1)

^a^Resultados com dados da Pesquisa Nacional de Demografia e Saúde da Criança e da Mulher (PNDS) 2006 (n=8.023); ^b^Resultados com dados da Pesquisa Nacional de Saúde (PNS) 2013 (n=9.994); ^c^Resultados com dados da PNS 2019 (n=10.774); ^d^Estimativas populacionais; ^e^Faixa etária utilizada para análise com dados da PNDS 2006 e da PNS 2019; ^f^Faixa etária utilizada com dados da PNS 2013.

**Tabela 3:** Prevalência (%) e intervalo de confiança de 95% (IC95%) de laqueadura tubária em todas as mulheres brasileiras em idade reprodutiva, de acordo com as características sociodemográficas. Brasil, 2006, 2013 e 2019

Características sociodemográficas	2006^a^	2013^b^	2019^c^
	%^d^ (IC95%)	%^d^ (IC95%)	%^d^ (IC95%)
Total	32,2 (30,6; 33,9)	22,0 (20,8; 23,2)	17,3 (16,4; 18,2)
**Faixa etária** (anos)			
15-19^e^	0,0 (0,0; 3,5)	-	0,0 (0,0; 0,2)
18-19^f^	-	0,3 (0,0; 1,7)	-
20-24	3,5 (2,5; 5,0)	1,9 (1,2; 3,1)	1,5 (1,0; 2,3)
25-29	17,4 (14,8; 20,3)	8,4 (7,0; 10,0)	8,1 (5,7; 11,4)
30-34	34,0 (30,4; 37,8)	19,9 (17,7; 22,3)	15,3 (13,6; 17,2)
35-39	47,6 (43,6; 51,6)	32,5 (29,6; 35,5)	23,4 (21,2; 25,7)
40-44	51,7 (46,7; 56,6)	40,1 (36,9; 43,5)	30,0 (27,4; 32,7)
45-49	68,0 (63,7; 72,1)	47,1 (42,4; 52,0)	37,1 (33,4; 40,9)
**Raça/cor da pele**			
Branca	26,2 (24,0; 28,6)	16,1 (14,7; 17,7)	12,9 (11,6; 14,3)
Preta/parda	37,1 (34,9; 39,3)	27,2 (25,6; 28,9)	20,6 (19,3; 21,9)
Escolaridade			
Ensino fundamental	43,4 (41,0; 45,9)	36,8 (34,5; 39,0)	30,8 (29,7; 23,9)
Ensino médio	21,2 (19,3; 23,3)	15,5 (14,0; 17,0)	14,8 (13,5; 16,1)
Ensino superior	18,1 (15,0; 21,7)	11,7 (10,0; 13,6)	9,2 (8,0; 10,5)
Região			
Centro-Oeste	43,4 (40,4; 46,4)	30,1 (27,2; 33,1)	22,8 (20,1; 25,6)
Norte	47,8 (44,2; 51,4)	31,9 (28,8; 35,1)	27,9 (25,3; 30,5)
Nordeste	43,9 (40,4; 47,5)	33,8 (31,6; 36,1)	25,7 (24,1; 27,3)
Sudeste	25,2 (22,5; 28,1)	15,8 (13,9; 18,0)	12,1 (10,6; 13,7)
Sul	20,9 (18,3; 23,8)	9,1 (7,3; 11,4)	8,7 (7,3; 10,4)
**Local da residência**			
Urbana	30,7 (29,0; 32,6)	20,4 (19,2; 21,7)	16,0 (15,0; 17,0)
Rural	40,0 (35,6; 44,5)	33,0 (29,8; 36,4)	26,8 (24,4; 29,3)
**Posse de plano de saúde**			
Não	36,3 (34,5; 38,2)	25,3 (23,9; 26,7)	20,0 (19,0; 21,1)
Sim	21,9 (19,1; 25,0)	14,6 (12,7; 16,7)	10,2 (8,9; 11,7)
**Número de partos**			
Nenhum	1,0 (0,3; 3,5)	4,6 (1,7; 11,6)	3,5 (1,8; 6,8)
1	4,0 (2,9; 5,5)	1,7 (1,3; 2,3)	2,6 (2,0; 3,3)
2	43,8 (40,8; 46,9)	29,9 (27,6; 32,3)	24,1 (22,2; 26,0)
3	64,2 (60,2; 68,1)	54,0 (50,1; 57,8)	44,1 (40,3; 48,0)
4+	27,4 (24,4; 30,6)	17,7 (16,0; 19,5)	48,0 (44,0; 51,9)

^a^Resultados com dados da Pesquisa Nacional de Demografia e Saúde da Criança e da Mulher (PNDS) 2006 (n=11.324); ^b^Resultados com dados da Pesquisa Nacional de Saúde (PNS) 2013 (n=14.394); ^c^Resultados com dados da PNS 2019 (n=16.640); ^d^Estimativas populacionais; ^e^Faixa etária utilizada para análise com dados da PNDS 2006 e da PNS 2019; ^f^Faixa etária utilizada com dados da PNS 2013.

Mulheres que residiam nas regiões Sul (2006 – 20,9%; 2013 – 9,1%; 2019 – 8,7%) e Sudeste (2006 – 25,2%; 2013 – 15,8%; 2019 – 12,1%) apresentaram menores prevalências de laqueadura (Tabelas 3 e 4). Observou-se redução da prevalência de laqueadura, segundo essas características, entre 2006 e 2019, exceto para o grupo de mulheres que tiveram quatro ou mais partos, com aumento de 75,2% para o grupo de todas as mulheres (Tabela 3). Isso não foi observado para o grupo de mulheres que moravam com companheiro (Tabela 4).

## Discussão 

Estes achados revelaram aumento da prevalência de vasectomia dos parceiros referida pelas mulheres e redução da laqueadura tubária entre 2006 e 2019, embora a prevalência de métodos cirúrgicos entre homens permanecesse inferior à das mulheres. Mulheres com maior vulnerabilidade social, como pretas e pardas, com menor escolaridade, de áreas rurais e sem plano de saúde, apresentaram maiores prevalências de laqueadura tubária. Mulheres com melhores condições sociodemográficas, como maior escolaridade, residentes em áreas urbanas, nas regiões Sul e Sudeste e com acesso a plano de saúde, tiveram maiores prevalências de parceiros que fizeram vasectomia. Esses resultados revelaram a persistência de desigualdades sociais no acesso aos métodos contraceptivos permanentes.

Como limitações deste estudo, a vasectomia foi referida pelas mulheres sobre seus companheiros, o que pode configurar uma desigualdade de gênero das próprias pesquisas sobre o tema. Os dados de inquéritos demográfico de 1986 e da PNDS em 1996 não foram incluídos devido à falta de comparabilidade amostral. Se incluídos, seria observado aumento contínuo da vasectomia, semelhante ao observado em 2006, 2013 e 2019 (7,8). Para laqueadura tubária, seria observado aumento entre 1986 e 1996 e depois diminuição observada no período estudado (7,8). 

Outra limitação referiu-se à comparabilidade das variáveis faixa etária e escolaridade. No entanto, para essa última, considerou-se a equivalência entre classificação da escolaridade e anos de estudo. Também não foi possível realizar análise de tendência, por ter apenas três medidas durante o período, mas foi possível observar a evolução desses indicadores ao longo do tempo, destacando-se a necessidade desse monitoramento para subsidiar políticas públicas na área da saúde sexual e reprodutiva.

Na França, entre 2010 e 2022, houve aumento de vasectomia, com maior uso desse método por homens entre 41 e 44 anos e que possuíam melhores condições socioeconômicas (21), corroborando este achado. Para laqueadura tubária, ocorreram aumento entre 2010 e 2013 e redução entre 2013 e 2022 para mulheres com condições socioeconômicas menos favoráveis (21). Esse cenário mostrou que o acesso aos métodos contraceptivos permanentes também apresenta iniquidades em países desenvolvidos. Entretanto, na França, entre 2021 e 2022, foram realizadas mais vasectomias que laqueaduras (21), realidade distinta a do Brasil. 

O índice de empoderamento das mulheres é um medidor com dez indicadores, entre eles acesso aos métodos contraceptivos modernos, incluindo os de longa duração. Por meio dele, o Brasil apresentou médio-baixo empoderamento (0,637), atrás dos Estados Unidos (0,752) e do Reino Unido (0,778), ambos médio-alto empoderamento (22). Percebe-se que o empoderamento das mulheres brasileiras ainda está em construção, o que reflete o acesso aos contraceptivos de longa duração, utilizados por apenas 5% das brasileiras (12,13). 

Tal fato poderia explicar a opção pela laqueadura tubária para mulheres que já alcançaram o número desejado de filhos devido à sua alta eficácia. Porém, ressalta-se a importância de aconselhamento profissional que promova uma escolha livre e informada, visto que o desejo de ter mais filhos pode mudar ao longo da vida, e a reversão da laqueadura não é amplamente acessível e tem baixa taxa de sucesso (1).

O índice de desigualdade de gênero é constituído por indicadores de saúde reprodutiva, empoderamento e mercado de trabalho e tem relação com o uso dos métodos contraceptivos permanentes. A exemplo, a prevalência de vasectomia em 2019 nos Estados Unidos foi 14% e no Brasil foi 5%, enquanto a prevalência de laqueadura tubária foi 19,0% e 21,8%. Conforme o índice de desigualdade de gênero, a classificação desses países é 12º (Estados Unidos) e 31º (Brasil) (23). Quanto maior for o índice de desigualdade de gênero, menos evidente é o papel do homem no planejamento reprodutivo. 

Globalmente, as mulheres utilizam mais os métodos contraceptivos que os homens, o que reforça a desigualdade de gênero no âmbito da contracepção. Em 2019, no Brasil, a prevalência do uso de preservativo masculino foi 20%, enquanto o uso de contraceptivo oral foi 40% (12). Essa disparidade se agrava com a maior disponibilidade de contraceptivos para as mulheres (preservativo feminino, diafragma, dispositivo intrauterino, pílulas, injetáveis, implantes), enquanto há apenas o preservativo masculino e a vasectomia para os homens. 

Métodos masculinos têm sido investigados e ensaios clínicos foram abandonados devido a ocorrências de efeitos colaterais, como depressão e alterações da libido (24). Outros têm se concentrado em métodos orais e tópicos visando aumentar a praticidade do uso (24). Caso se mostrem eficazes e bem tolerados, têm potencial para utilização clínica. Porém, persistem barreiras ao desenvolvimento de contraceptivos masculinos hormonais, como ausência de investimento, preocupações com os efeitos colaterais, efeito rebote espermatogênico, falta de reversibilidade comprovada e eficácia dos métodos não hormonais (25). Esses desafios reforçam a persistência das desigualdades de gênero, e a mulher continua sendo a protagonista do planejamento reprodutivo. 

Políticas do Ministério da Saúde voltadas à saúde do homem (26,27) tiveram, entre seus objetivos, estimular e ampliar a realização de vasectomia, por meio da implementação e expansão do sistema de Atenção à Saúde do homem (27). Isso pode ter contribuído para o aumento da prevalência de vasectomia encontrado no período avaliado. Outras possíveis explicações para esse achado incluíram: maior conhecimento sobre a segurança e eficácia da vasectomia entre os homens; aumento da participação do homem na decisão sobre o uso de contracepção; maior proporção de casais que desejam ter um número menor de filhos ou não desejam ter filhos, ou que já alcançaram o número desejado de filhos; dificuldades econômicas para criação dos filhos; e maior conhecimento do parceiro em relação à laqueadura tubária ser mais propícia a intercorrências do que a vasectomia (28).

As hipóteses para a baixa prevalência de vasectomia no país foram dadas em virtude de fatores como: persistente visão patriarcalista de que a contracepção é responsabilidade da mulher; falta de conhecimento sobre esse método contraceptivo; baixa procura dos homens por serviços de saúde; e preconceito e medo acerca da ideia errônea de que a vasectomia causará impotência (23,29). Esse contexto de informações precárias pode levar à perpetuação da desigualdade de gênero e contribuir para as desigualdades sociodemográficas também observadas na prevalência desses métodos.

As desigualdades sociodemográficas podem ser pelo uso de cada um dos métodos cirúrgicos por grupos distintos. Enquanto foram observadas maiores prevalências de laqueadura entre mulheres com piores condições sociodemográficas como menor escolaridade, pretas e pardas, sem acesso a plano de saúde, residentes em áreas rurais e com maior paridade, aquelas com melhores condições, como residir em áreas urbanas e nas regiões Sul e Sudeste do país, ter plano de saúde e maior escolaridade, relataram maiores prevalências de vasectomias em seus parceiros. 

Este estudo reforçou as desigualdades existentes no tipo de contraceptivo utilizado pelas brasileiras, conforme estudos prévios (12,13). No entanto, avançou ao mostrar essa diferença entre a laqueadura tubária e a vasectomia, apontando para a necessidade de ampliação do acesso à vasectomia para a população com maior vulnerabilidade social.

Mulheres com maior vulnerabilidade social enfrentam mais obstáculos para obtenção de informações sobre os métodos contraceptivos, e como acessá-los, o que pode acarretar menor poder de decisão sobre qual método usar. A escolaridade é um dos principais determinantes do uso de contraceptivos, uma vez que possibilita maior acesso à informação e ao conhecimento, além de se constituir em veículo de mobilidade socioeconômica e autonomia sexual e reprodutiva (30), o que contribui para explicar as desigualdades observadas. 

Autonomia sexual e reprodutiva diz respeito a direitos reprodutivos, escolha do momento de engravidar, quantidade desejada de filhos e tomada de decisão sobre o uso do método contraceptivo (31). Considerando as iniquidades de acesso aos métodos contraceptivos a que essas mulheres estão submetidas, pode-se concluir que elas estão sendo privadas de sua plena autonomia, evidenciando necessidade de avançar em acesso mais equitativo ao planejamento reprodutivo no país.

Em 2019, aproximadamente metade das mulheres brasileiras utilizou métodos hormonais (12). Somente em 2013, a laqueadura tubária deixou de ser o método contraceptivo mais utilizado pelas brasileiras (13). Essa realidade reafirma que o uso do método está relacionado com as informações recebidas pelas brasileiras e suas oportunidades de acesso. 

Os contraceptivos de longa duração, métodos mais eficazes, são mais utilizados pelas mulheres que possuem maior renda, escolaridade e plano de saúde (12), assim como observado para vasectomia, reforçando a importância do maior acesso ao método contraceptivo e às informações sobre ele para se alcançar a justiça contraceptiva. Logo, a atenção a ser oferecida à população quanto à anticoncepção deve incluir informações sobre indicação, modo de usar, efeitos adversos, vantagens e desvantagens, até o acompanhamento clínico regular, tornando possível proporcionar qualidade de vida, saúde e dignidade reprodutiva, além de maior autonomia na escolha de seu método contraceptivo (32). 

Os achados deste estudo, de aumento da prevalência da vasectomia e redução da laqueadura tubária entre 2006 e 2019, representaram avanço na saúde sexual e reprodutiva da população brasileira, resultado do maior acesso a outras opções de métodos contraceptivos. Por outro lado, constatou-se a persistência de desigualdades de gênero e sociodemográficas no acesso aos métodos permanentes no país. 

Os retrocessos das políticas públicas voltadas para garantia da esterilização cirúrgica masculina e feminina no SUS, no âmbito da Saúde Sexual e Reprodutiva (13), tais como o conservadorismo crescente na sociedade brasileira (33), podem dificultar ainda mais a participação masculina na contracepção. Reconhece-se que, nos próximos inquéritos populacionais, os resultados podem ser diferentes, visto que, durante a pandemia de covid-19, as cirurgias eletivas foram interrompidas (34). Isso pode ter influenciado na redução do uso dos métodos permanentes, apesar das mudanças na legislação que facilitaram o acesso, com a exclusão da autorização do cônjuge, um avanço na luta pela igualdade de gênero. 

Estudos futuros serão necessários para seguir monitorando essa evolução temporal. O contínuo monitoramento desses indicadores de uso de método contraceptivo no país poderia direcionar, com maior efetividade, investimentos em acesso mais qualificado e equitativo, considerando principalmente a necessidade de cada indivíduo e respeitando o exercício dos direitos sexuais e reprodutivos.
